# Human germline editing: Insights to future clinical treatment of diseases

**DOI:** 10.1007/s13238-018-0594-8

**Published:** 2018-11-14

**Authors:** Yanni Li, Xiang Jin Kang, Jeremy Kah Sheng Pang, Boon Seng Soh, Yang Yu, Yong Fan

**Affiliations:** 1 Key Laboratory for Major Obstetric Diseases of Guangdong Province, Center of Reproductive Medicine, The Third Affiliated Hospital of Guangzhou Medical University, Guangzhou, 510150 China; 2 Disease Modeling and Therapeutics Laboratory, A*STAR Institute of Molecular and Cell Biology, 61 Biopolis Drive Proteos, Singapore, 138673 Singapore; 3 Center of Reproductive Medicine, Department of Obstetrics and Gynecology, Peking University Third Hospital, Beijing, 100191 China

Last year, the first attempt to genetically modify human embryos in the United States was reported and sparked a huge debate (Ma et al., 2017). Although the first human germline modification was only performed two years ago, the study showed that rapid advances in technology has allowed the rate of off-target effects and mosaicism to be reduced considerably (Liang et al., 2015). Recently, Vertex and CRISPR therapeutics collaborated and developed CTX001, the first CRISPR/Cas9-based therapy, targeting patients with β-thalassemia and have begun phase 1/2 clinical trials. With policies and technologies regarding genome editing both developing rapidly, explorations into the possibility of clinical gene editing for hundreds of hereditary diseases are starting to become achievable. Here, we address the progress of human embryo editing technologies so far and its promise and risks in advancing therapy for hereditary diseases.

Researchers have utilized genome editing techniques to modify genetic sequences in somatic cells and germline cells to conduct basic research on gene function or disease treatment. Genetic modifications to a somatic cell are generally non-heritable as they do not contribute to gametes. However, researchers have utilized tetraploid complementation to produce genetically modified offspring from modified mouse and rat pluripotent stem cells (Eggan et al., 2002; Li et al., 2017c). On the other hand, genetic editing to an organism’s germ cells is more universally applicable and will result in the natural inheritance of the modified genome in its offspring. As current genome editing technology often introduces off-target effects such as chromosomal translocations or insertion-deletions (indels) resulting in undesired loss or gain of functions of genes, which is a safety concern when dealing with the human germline (Corrigan-Curay et al., 2015), the potential of genome editing to overcome genetic diseases is therefore held back by the risk of creating more genetic complications or even irreversibly altering the human germline through nondescript mutations.

Genome editing research is rather commonplace nowadays, with CRISPR/Cas9-mediated genome modification being at the forefront since its first adaptation into eukaryotic cells (Abrahimi et al., 2015; Cao et al., 2016; Cong et al., 2013; Hsu et al., 2014; Iyer et al., 2018; Li et al., 2017b; Mali et al., 2013; Nelson et al., 2016; Noel et al., 2018; Sato et al., 2015; Savic et al., 2018; Schwank et al., 2013; Shalem et al., 2014; Shen et al., 2014; Slaymaker et al., 2016; Wu et al., 2013). The diverse amounts of *ex vivo* and *in vivo* experiments conducted have resulted in the genome editing protocol to be significantly improved in the last 5 years. Today, nuclease delivery into cells for genome editing can be either in the form of RNA or protein for enhanced kinetics of action and nuclease turnover, while also preventing integration of exogenous DNA into the host genome (Abou-El-Enein et al., 2017). Efficient and precise gene correction for mutations takes advantage of the cell cycle, relying on the homology-directed repair (HDR) pathway which functions in the late S–G_2_ phase (Chapman et al., 2012; Heyer et al., 2010). In the recent year, germline editing has become a hot topic in scientific research. CRISPR/Cas9 microinjections into mouse zygotes have been shown to correct disease associated mutations, producing healthy adult animals (Wang et al., 2013; Wu et al., 2013). Likewise, CRISPR/Cas9 and TALEN have both effectively generated germline mutations in fertilized monkey embryos (Liu et al., 2014; Niu et al., 2014). With numerous successes in both somatic and germline genome editing in animal models, researchers have started to consider the possibility of translating the protocol to edit the human genome for clinical purposes (Cornu et al., 2017).

For the first time, US National Academy of Sciences and National Academy of Medicine relaxed stance on modification of germline in February 2015 followed by the first reports of gene editing in human embryos. Tripronuclear (3PN) zygotes which were discarded from clinics were used by a group in China to attempt CRISPR/Cas9-mediated gene editing of human endogenous β-globin gene (*HBB*). They found that the efficiency of single-stranded oligonucleotides (ssODNs) mediated HDR was low and successfully edited embryos exhibited mosaicism i.e., only a portion of the cells were successfully modified, while the remaining cells remained as wild type (Liang et al., 2015). In addition, whole-exome sequencing revealed off-target mutations in these 3PN embryos. Moreover, the researchers identified that although the endogenous delta-globin gene (*HBD*), a *HBB* homolog, also functions as a template to compete with ssODNs for HDR repair, majority of the double-stranded breaks (DSBs) caused by Cas9 were repaired through the unideal error-prone non-homologous end joining (NHEJ) pathway.

Although the efforts of Liang et al. were not a complete success, it was the first attempt in modifying the human genome and caught the attention of the international stage. A group of 22 experts at the International Summit in December 2015 convened to discuss the science, ethics and governance of human genome editing. The report supported basic research to uncover knowledge regarding early human embryo development and heritable diseases, and concluded that within a set of compelling conditions, germline editing would be ethically defensible and allowed. In addition, a list of guidelines were set for any country seeking to engage in human germline editing research, and strongly suggested to seek public opinion on policies regarding germline editing (Pei et al., 2017).

**Figure 1:**
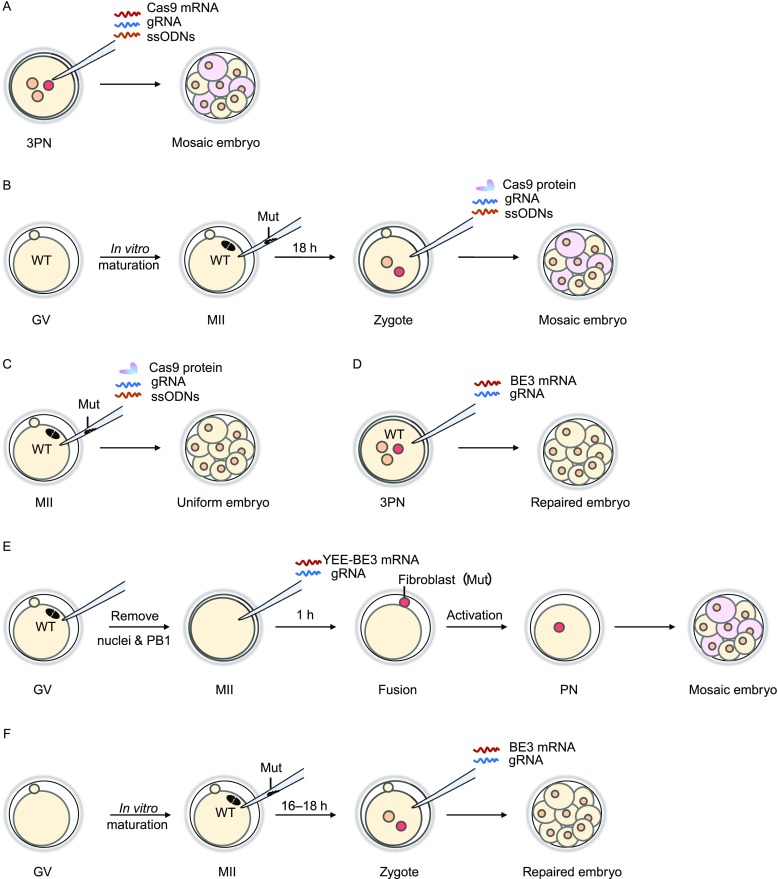
**Schematic of human embryo gene editing**. (A) Schematic of introducing mutation into *HBB* and *CCR5* gene in clinical discarded 3PN embryos by CRISPR/Cas9 component. (B) Schematic of CRISPR/Cas9-mediated gene correction of β41–42 (-TCTT) mutation in *HBB* and G1376T mutation at the X-linked *G6PD* locus in human 2PN zygotes. (C) Schematic of *MYBPC3*^ΔGAGT^ gene targeting by Cas9 protein/gRNA/ssODNs were co-injected with sperm into MII oocytes during intracytoplasmic sperm injection (ICSI). This system allows the elimination of mosaicism. (D) Schematic of introducing point mutation into *HBB*, *FANCF* and *DNMT3B* gene or *HEK293* site 4 and *RNF2* gene in human 3PN embryos. Base editor shows highly efficient in gene editing, but haven’t performed blastomeres sequencing to detect mosaicism. (E) Schematic of repairing *HBB* −28 (A>G) in cloned human embryos by base editing system. Human homozygous *HBB* −28 (A>G) mutant embryos were constructed by fusing skin fibroblast cell from the patient with *in vitro* matured. The 1st polar body (PB1) and nucleus were removed before fusing. (F) Schematic of correcting *FBN1*^T7498C^ mutation by BE3 in heterozygous mutant embryos

In 2016, we tried to introduce the naturally occurring beneficial *CCR5*Δ32 allele into early human 3PN embryos by CRISPR/Cas9 (Fig. 1A). Although the HDR efficiency was low and the edited embryos exhibited mosaicism, we did not detect any mutation from 28 potential off-target sites (Kang et al., 2016). Due to worries regarding low HDR efficiencies, mosaicism and the need for more specific assays to identify off-target mutagenesis, germline editing was forbidden on any embryos intended for *in vitro* fertilization and implantation by international scientists, ethicists, legal experts and patient groups from around the world on 14 February 2017. The American Society of Human Genetics (ASHG) workgroup addressed ethical principles, scientific boundaries and policy issues, published in *The American Journal of Human Genetics* to guide germline editing research. The proposition similarly disapproves of germline editing that culminates in pregnancy, but supports *in vitro* germline editing to facilitate possibly future applications in the clinical field to treat or prevent diseases (Ormond et al., 2017).

Following Kang et al.’s success in achieving gene editing in human 3PN embryos, Tang et al. published their attempt in assessing the efficiency of CRISPR/Cas9 in correcting disease-causing mutations directly in clinical-quality human embryos in March 2017. Wild-type donated oocytes were subjected to *in vitro* maturation and fertilization by intracytoplasmic injection with disease-causing mutation β41–42 (-TCTT) in *HBB* sperms (Fig. 1B). They demonstrated that CRISPR/Cas9 system is proficient in correcting point mutations in human zygotes with increased HDR efficiency of *HBB*. However, mosaicism and off-target effects were still observed (Tang et al., 2017).

The three previously discussed studies all obtained genetically mosaic embryos (Kang et al., 2016; Liang et al., 2015; Tang et al., 2017). Ma et al. demonstrated an effective CRISPR/Cas9 protocol, which takes advantage of a DNA repair mechanism unique to early embryos to correct a disease-causing gene in the germline. By co-injection of sperm, Cas9 protein, gRNA and ssODNs into metaphase II (MII) oocytes, they corrected a mutated heterozygous *MYBPC3* gene known to cause hypertrophic cardiomyopathy (HCM) in human preimplantation embryos with high precision, accuracy and efficiency (Fig. 1C), without generating large deletions (Adikusuma et al., 2018; Egli et al., 2018; Ma et al., 2018). They were able to show that HDR using the maternal WT DNA from the oocyte as the template to repair DSB induced in the mutant paternal DNA from the sperm was highly effective, and more dominant than the ssODNs supplied as a template. By utilizing the unique zygotic DNA repair mechanism, mosaicism and off-target mutations can be significantly reduced or eliminated entirely (Ma et al., 2017). Other researchers have shown similar results using human and mouse embryos (Liang et al., 2015; Wilde et al., 2018; Wu et al., 2013). However, the mechanism of zygotic DNA repair mechanism is currently not well understood, and there is also a need to develop a method to accurately restore gene mutants present in both parental DNA.

Base editing systems which utilize CRISPR technology to edit individual bases without inducing DSBs have been developed to treat the numerous heritable genetic diseases that arise from single nucleotide point mutations. Base editors (BE) are composed of a cytidine deaminase such as apolipoprotein B editing complex (APOBEC) or activation-induced deaminase (AID), gRNA, Cas9 nickase and uracil DNA glycosylase inhibitor (UGI). BE was developed to trigger a series of chemical reactions that ultimately substitute a C to T (or G to A) at a target site within a window of approximately five nucleotides (Komor et al., 2016; Tang et al., 2017). BE has been shown to be effective in plants (Chen et al., 2017; Lu and Zhu, 2017), yeast (Zong et al., 2017), human cells and mouse embryos with 100% point mutation efficiency without any mosaicism detected (Kim et al., 2017a, b; Liang et al., 2017b).

Two groups have employed base editing system to study single-nucleotide mutation in 3PN human embryos independently (Fig. 1D). Zhou et al. used BE3 to introduce a point mutation into human *HBB* gene and modified SaKKH-BE3, with a more relaxed PAM requirement and broader genome targeting scope, to target *FANCF* and *DNMT3B* gene. Both methods showed efficient and precise base editing in human 3PN embryos. In addition, screening for off-target effects showed that only 1 off-target mutation occurred out of 1,187 potential off-target sites (Zhou et al., 2017). A separate study by another group targeted two human gene sites, *HEK293* site 4 and *RNF2*, to evaluate the efficiency of base editing. They were able to achieve high base editing efficiency with none of the associated drawbacks of on-target indels, off target mutagenesis and C-G/C-A unwanted base substitutions (Li et al., 2017a).

To demonstrate the applicability of base editors in 2PN embryos, Liang et al. constructed human embryos by fusing *in vitro* matured oocytes with their 1st polar body and spindle removed, together with skin fibroblast cells from peripheral blood, both from the same *HBB* −28 (A>G) mutant patient. They injected YEE-BE3, a BE3 variant with a smaller deamination window together with gRNA into the enucleated oocytes before fusion with the fibroblast (Fig. 1E). The mutation repairing efficiency was estimated to be around 20% and no off-target deamination was found. However, most of the blastomeres in repaired embryos were mosaic and minor G-A/G-C mutations were found (Liang et al., 2017a). More recently, a research team successfully corrected a Marfan syndrome pathogenic mutation using base editing in human cells and zygotic embryos (Fig. 1F). Precise base substitution was achieved without detection of any off-target or indels. However, undesired base conversion events were detected and blastomere sequencing was not conducted to test for mosaicism (Zeng et al., 2018). To that end, these studies were able to demonstrate the potential of base editing systems in treating point mutation disease in 2PN embryos.

The CRISPR/Cas9 systems have been rapidly modified and improved by researchers in an effort to reduce the potential downsides and riskiness of genome editing therapy. We have demonstrated the protocol that takes advantage of the zygotic DNA repair response unique to early embryos which preferentially repairs DNA DSBs using endogenous homologous sequence, making it highly applicable for heterozygous mutant treatment (Ma et al., 2017). Also, several groups experimenting with base editing technology have also shown its effectiveness in inducing base substitutions without introducing DSBs (Gaudelli et al., 2018; Komor et al., 2017). As long as there is a potential of off-target effects, germline mutations are risky as unintended mutations introduced into the human genome could be inherited by the next generation. Mosaicism in edited embryos present another crucial problem as it would invalidate gene correction therapy, having some uncorrected blastomeres contribute to the adult phenotype and potentially the germline. As such, one potential issue with mosaicism is that it hampers the possibility of predicting gene editing outcomes through pre-implantation genetic diagnosis (PGD) for clinical applications. Another concern pertains to the identification of potential toxicities from *ex vivo* gene-edited cellular products. There has been reports of CRISPR/Cas9 achieving HDR from a donor template by inhibition of p53 pathway, which suggests the risk of disturbing the crucial function of *p53* in edited cell lines (Haapaniemi et al., 2018). Classical gRNA guided CRISPR/Cas9 nuclease activity involves DSBs, frequently resolving into large indels, translocations and inversions, potentially bringing about pathogenic lesions (Kosicki et al., 2018). Therefore, there is a need to develop the genome editing technology further in efficiency and safety before considering therapeutic options and germline editing of human embryos.

Since 2015, several nations including China, US, UK and Sweden have authorized genome editing on human embryos. Lately, the Nuffield Council on Bioethics, a leading UK ethics body, considered the DNA editing of a human embryo meant for eventual birth morally permissible if it is for the child’s interest and has no ill-effects on society. Concerns of germline editing revolve around non-therapeutic treatments, such as the creation of “designer babies”. Therefore, it is important to set universally adhered guidelines that researchers follow strictly regarding the ethics of germline experimentation, such as to consider the interests of humanity. For now, any attempts to generate genetically modified humans through early embryo editing are prohibited until we can overcome the ethical and scientific challenges.
